# The effects of Secondhand Smoke (SHS) exposure on microvascular endothelial function among healthy women

**DOI:** 10.1186/s12971-015-0052-9

**Published:** 2015-09-03

**Authors:** Zulkefli Sanip, Siti Hajar Mohd Hanaffi, Imran Ahmad, Siti Suhaila Mohd Yusoff, Aida Hanum Ghulam Rasool, Harmy Mohamed Yusoff

**Affiliations:** Central Research Laboratory, School of Medical Sciences, Universiti Sains Malaysia, Kubang Kerian, Kelantan Malaysia; Department of Family Medicine, School of Medical Sciences, Universiti Sains Malaysia, Kubang Kerian, Kelantan Malaysia; Department of Pharmacology, School of Medical Sciences, Universiti Sains Malaysia, Kubang Kerian, Kelantan Malaysia; Faculty of Medicine, Universiti Sultan Zainal Abidin, City Campus, 20400 Kuala Terengganu, Terengganu Malaysia

## Abstract

**Background:**

Studies have demonstrated that secondhand smoke (SHS) exposure could impair endothelial function. However, the effect of SHS exposure specifically on microvascular endothelial function is not well understood. This study aimed to determine the effects of SHS exposure on microvascular endothelial function among non-smoking, generally healthy women.

**Findings:**

We studied 127 women; and based on their hair nicotine levels measured using gas chromatography-mass spectrometry, 25 of them were categorized as having higher hair nicotine levels, 25 were grouped as having lower hair nicotine and 77 women were grouped into the non-detected group. The non-detected group did not have detectable levels of hair nicotine. Anthropometry, blood pressure (BP), lipid profile and high-sensitivity C-reactive protein (hsCRP) were measured accordingly. Microvascular endothelial function was assessed non-invasively using laser Doppler fluximetry and the process of iontophoresis involving acetylcholine and sodium nitroprusside as endothelium-dependent and endothelium-independent vasodilators respectively. The mean hair nicotine levels for higher and lower hair nicotine groups were 0.74 (1.04) and 0.05 (0.01) ng/mg respectively. There were no significant differences in anthropometry, BP, lipid profile and hsCRP between these groups. There were also no significant differences in the microvascular perfusion and endothelial function between these groups.

**Conclusion:**

In this study, generally healthy non-smoking women who have higher, lower and non-detected hair nicotine levels did not show significant differences in their microvascular endothelial function. Low levels of SHS exposure among generally healthy non-smoking women may not significantly impair their microvascular endothelial function.

## Background

Endothelial dysfunction is one of the earliest vascular changes that occur in the pathogenesis of many cardiovascular diseases (CVD) [1] including the development of atherosclerosis [2]. Reversibility of endothelial dysfunction has been demonstrated with various pharmacological interventions such as statins, angiotensin-converting enzyme inhibitors (ACEI), and metformin [3–5]. Microcirculation is said to be the initial site of endothelial damage for women who are at risk of CVD [6]. Therefore, the assessment of microvascular endothelial function can be utilized as a tool to detect early vascular changes [7] that may occur due to medical conditions and diseases, or to monitor the response to pharmacological interventions.

According to the Surgeon General’s report in 2010, the exposure to SHS leads to a rapid and sharp increase of endothelial dysfunction and inflammations, which are implicated in acute cardiovascular events and thrombosis [8]. There were fast accumulating evidences of cardiovascular related parameters which include platelet and endothelial function, arterial stiffness, atherosclerosis, inflammation, oxidative stress, heart rate variability, energy metabolism, and increased infarct size, being delicately responsive to the toxins in SHS [9]. Early studies have showed that cigarette smoke contains lipophilic substances that are toxic to bovine arterial endothelial cells [10], and can directly damage the integrity of the endothelial cell layer in man and reduce vasodilator properties in vitro [11]. Therefore, we hypothesized that there may be differences in microvascular endothelial function among generally healthy non-smoking women who were exposed and non-exposed to SHS. Women with detectable levels of hair nicotine were further divided in those with higher and lower levels of high nicotine; differences in microvascular endothelial function between the three groups were studied.

## Methods

### Subjects

SHS is defined as combination of side-stream cigarette smoke (the smoke given off from a burning tobacco product) and the exhaled mainstream smoke [12]. In this comparative cross-sectional study, we divided the subjects into three groups based on their hair nicotine levels; which were groups with higher hair nicotine levels (*n* = 25), lower hair nicotine levels (*n* = 25) and non-detected group (*n* = 77). The number of subjects in higher and lower hair nicotine groups were divided equally based on their hair nicotine rank. All subjects were generally healthy with serum low-density lipoprotein cholesterol (LDL-C) levels below 5.0 mmol/l, non-smokers, normotensive, non-diabetic (fasting blood sugar (FBS) below 6.1 mmol/l), not on routine medications, and possess no history of acute or chronic diseases such as CVD, renal, liver, haematological diseases, or active psychiatric conditions. The study protocol has been approved by Research Ethics Committee (Human) of Universiti Sains Malaysia and all subjects have signed the informed consent before any procedures were performed. Subjects were required to refrain from consuming any beverages containing caffeine and salty meal starting a day before the test and fast for at least 10 h before the vascular examination.

### Anthropometry, blood pressure, blood analysis and hair nicotine levels

Body weight and height were measured using a digital weighing scale attached with stadiometer (SECA, Hamburg, Germany). Body mass index (BMI) was calculated as the ratio of weight (kg) to height (m^2^) (kg/m^2^). Systolic and diastolic blood pressure (SBP and DBP) were measured on the right arm in sitting position using a digital blood pressure monitor (MX3, Omron, Japan) after at least 10 min of resting. Two readings were taken with 5 min apart and the mean reading was recorded. Fasting venous blood was taken for lipid profile [total cholesterol (TC), triglyceride (TG), low-density lipoprotein cholesterol (LDL-C), high-density lipoprotein cholesterol (HDL-C)] and hsCRP measurement. Hair samples were obtained for hair nicotine level analysis as described in previous study [13]. The hair samples were cut as close as possible to the scalp and only 10 cm (maximum length) of hair samples (measured from the scalp end) were used for hair nicotine analysis. Generally, the procedures for hair nicotine analysis involve hair washing, digestion, extraction and gas chromatography-mass spectrometry (GCMS) analysis while the limit of quantitation (LOQ) was 0.04 ng/mg.

### Assessment of microvascular endothelial function

The microvascular endothelial function was assessed non-invasively using laser Doppler fluximetry (LDF) and iontophoresis process; where the procedures and reproducibility had been reported previously [14, 15]. Iontophoresis is a method of delivering ionic drugs into the skin by applying low voltage to a drug solution. The local cutaneous forearm skin perfusion was measured by Dual-channel DRT4 LDF with DP1T-V2 skin laser probe (Moor Instruments, Axminster, United Kingdom) and permit real-time continuous measurement of microvascular perfusion. Iontophoresis of freshly prepared of 1 % ACh (Fluka Chemie Gmbh, Japan) and 0.9 % SNP (Riedel-de Haen, C.O.O. Switzerland) in sodium chloride (Excel Pharmaceutical, Selangor, Malaysia) were used to assess endothelial dependent vasodilatation and endothelial independent vasodilatation respectively.

Vascular measurements were conducted in the morning in a quiet room with a constant temperature. The subjects lie down in a supine position with the right forearm uncovered. Two iontophoresis chambers for 400 μL of ACh and SNP solutions were used simultaneously and the laser probes were fixed into the chambers. Two minutes of baseline perfusion were recorded before the measurement. ACh and SNP induced vasodilatation were quantified using these parameters; maximum change in perfusion (max), percent change in perfusion (percentage change), peak flux (peak), and area under the curve (AUC) as defined previously [14]. The blood perfusion was recorded as flux and expressed using arbitrary unit (AU). Intraday and interday coefficient of variations in this study for ACh_max_ was 11.6 and 12.5 % respectively.

Data was presented in terms of mean and standard deviation (SD). Mean comparison of anthropometry (weight, BMI), blood pressure (SBP, DBP), lipid profile (TC, TG, LDL-C, HDL-C), hsCRP and hair nicotine levels between higher, lower and non-detected groups were performed using one way analysis of variance (ANOVA). *p* value of less than 0.05 was denoted as significant.

## Results

The mean hair nicotine levels for higher and lower hair nicotine groups were 0.74 and 0.05 (0.01) ng/mg respectively. The levels of hair nicotine in higher hair nicotine group were significantly different with lower and non-detected hair nicotine groups. Table 1 showed no significant differences in the anthropometry, blood pressure, lipid profile and hsCRP between higher, lower and non-detected hair nicotine groups. Similarly, there were no significant differences in microvascular endothelial-dependent and endothelial-independent vasodilatations parameters (Table 1).

**Table 1:** Anthropometry, blood pressure, lipid profile, hsCRP and microvascular endothelial function of the three study groups

Parameters	Higher Hair Nicotine (*n* = 25)	Lower Hair Nicotine (*n* = 25)	Non-Detected (*n* = 77)	*F* stat. (df)	*p* Value
Hair Nicotine (ng/mg)	0.74 (1.04)	0.05 (0.01)	-	25.255 (2124)	0.000
Age (years)	33.92 (8.47)	31.92 (6.56)	35.01 (7.90)	1.512 (2124)	0.225
Weight (kg)	62.64 (12.05)	56.06 (14.36)	58.68 (11.12)	1.931 (2124)	0.149
BMI (kg/m^2^)	26.11 (4.68)	24.13 (4.94)	24.28 (4.10)	1.833 (2124)	0.164
SBP (mmHg)	116.10 (11.83)	111.08 (10.88)	113.14 (13.18)	1.026 (2124)	0.361
DBP (mmHg)	72.25 (7.19)	70.62 (8.00)	72.29 (9.20)	0.374 (2124)	0.689
Total Cholesterol (mmol/l)	5.25 (0.75)	5.57 (1.11)	5.47 (0.86)	0.833 (2124)	0.437
Triglyceride (mmol/l)	0.96 (0.29)	1.04 (0.37)	1.08 (0.48)	0.685 (2124)	0.506
LDL-C (mmol/l)	3.32 (0.83)	3.39 (0.84)	3.20 (0.87)	0.499 (2124)	0.608
HDL-C (mmol/l)	1.59 (0.28)	1.69 (0.47)	1.77 (0.38)	2.179 (2124)	0.117
hsCRP (mg/ml)	1.97 (3.23)	1.26 (2.55)	1.14 (2.07)	1.105 (2123)	0.334
ACh_max_ (AU)	44.42 (35.94)	58.73 (38.23)	41.00 (33.98)	2.398 (2124)	0.095
ACh Percent Change (%)	747.20 (519.81)	1163.91 (733.11)	824.42 (702.49)	2.934 (2124)	0.057
ACh Peak Flux (AU)	50.99 (37.33)	64.29 (39.38)	47.00 (35.52)	2.106 (2124)	0.126
Area Under Curve (AU x sec)	23683.29 (18330.72)	32733.11 (23516.35)	22831.67 (20934.55)	2.156 (2124)	0.120
SNP_max_ (AU)	56.69 (47.03)	71.57 (55.49)	54.61 (42.12)	1.306 (2124)	0.275
SNP Percent Change (%)	1212.80 (1107.81)	1453.16 (1110.19)	983.66 (680.17)	2.910 (2124)	0.058
SNP Peak Flux (AU)	62.08 (47.39)	77.11 (55.82)	61.71 (44.03)	1.057 (2124)	0.350
Area Under Curve (AU x sec)	32132.01 (27630.41)	35238.12 (27681.69)	30502.01 (25708.12)	0.305 (2124)	0.738

Data presented as Mean (SD)

One-way ANOVA

*BMI* body mass index, *SBP* systolic blood pressure, *DBP* diastolic blood pressure, *LDL-C* low density lipoprotein-cholesterol, *HDL-C* high density lipoprotein-cholesterol, *hsCRP* high sensitivity c reactive protein, *ACh*_*max*_ acetylcholine absolute change, *SNP*_*max*_ sodium nitroprusside absolute change

## Discussion

In the present study, there were no significant differences between the three groups for all parameters including microvascular endothelial function.

The endothelium plays a pivotal role in the regulation of vascular tone as well as in the maintenance of vascular integrity. In this respect, nitric oxide (NO) formed by endothelial cells is a crucial importance because it exerts a number of potential protective actions, including vasorelaxation and antithrombotic effects. A condition in which an impaired NO-mediated relaxation is associated with preserved vasodilation to an NO donor is often referred as endothelial dysfunction [16]. In the present study, we focused on the functional aspects of human peripheral microcirculation and studied its responses to ACh and SNP in the forearm skin among women exposed to SHS. However, exposure to SHS showed no significant differences in the responses of local microcirculation of both ACh and SNP.

It had been reported that acute high dose of SHS exposure for one hour did not damage the microvascular endothelial function among healthy non-smoker women [17]. Contrary, other findings showed significant reduction of skin blood flow reactivity, indicates the deterioration of peripheral microvascular endothelial function [18, 19]. We could not find a report on the effects of chronic SHS exposure to microvascular endothelial function. However, the effect of chronic SHS to endothelial function of larger blood vessels has been reported. A study reported that brachial-artery flow-mediated dilatation (FMD) was dose-dependently damaged in women with a history of SHS exposure for more than one hour daily for more than 3 years in comparison with the controls [20]. Another study stated that, healthy women who are exposed to SHS for more than 1 h daily for more than 10 years showed significant impairment of ACh-induced epicardial coronary artery dilatation, indicating endothelial dysfunction may occur diffusely to the subjects exposed to SHS [21].

The explanation for the lack of impairment of microvascular endothelial function in the current study remains unclear. Our study was designed to evaluate the effects of chronic exposure to SHS on microvascular endothelial function among healthy women. The intensity of exposure to SHS depends on a large number of variables, such as the exposure time per day, the proximity to the active smokers, the number of active smokers at home or workplace, and the size and ventilation of the rooms where SHS exposure occurs. One possibility to explain the lack of impairment of microvascular endothelial function in the current study may relate to the time of exposure. Our subjects may be exposed to SHS for short periods of time; subjects in previous studies which showed impairment of endothelial function in larger blood vessels were exposed to SHS for at least one hour per day [20, 21]. Another possibility is, most of our subjects were housewives, and the only active smokers were their husbands. Generally, they were not exposed to SHS while their husbands are at work and their main daily activities were also limited to house chores and close neighbourhood. The SHS exposure that they have might not be adequate to cause significant impairment in their microvascular endothelial function.

The heterogeneity of microvascular and macrovascular endothelial cells in respond to SHS exposure could be another reason why there were no significant changes in microvascular endothelial function in this study. Microvascular and macrovascular endothelial cells have been reported to show different responses to certain medical conditions or interventions. For example, coronary endothelial cells reacted with a decreased secretion of tissue-type plasminogen activator (tPA) and increase plasminogen activator inhibitor type 1 (PAI-1) activity to incubate with oxidized LDL-C, although there was only a minor response by the cardiac microvascular endothelial cells [22]. Besides, different microvascular and macrovascular responses with different stimulation have been demonstrated previously [23]. Macrovascular and microvascular perfusion increased significantly using induced hyperaemia at upper and lower arm. However, passive leg raising only changed the macrovascular indices significantly but not the microvascular indices. Moreover, among rheumatoid arthritis patients, there were no association observed between microvascular and macrovascular endothelial-dependent and independent functions [24], suggesting that microvascular and macrovascular have different regulations of their endothelial function.

Hair nicotine levels of SHS group in our study were comparatively lower compared to a few other previous studies [14, 25]. This may indicate that the exposure to cigarette smoke in our SHS subjects was relatively low which may contribute to the insignificant effect on microvascular endothelial function. An area that could be studied in the future includes the investigation on women who have heavier exposure to SHS, and confirming this exposure using objective measurement such as hair nicotine levels.

In this study, there were no significant differences in microvascular endothelial function between generally health non-smoking women with higher, lower and non-detected hair nicotine levels. The lack of microvascular endothelial function impairment in this study may be due to insufficient exposure to SHS. The reduction of endothelium-dependent vasodilatation of the skin microvasculature may become visible after many years of exposure, or when exposed to high intensity of SHS.

## Abbreviations

SHS: Secondhand smoke
GCMS: Gas chromatography-mass spectrometry
LDF: Laser Doppler fluximetry
ACh: Acetylcholine
SNP: Sodium nitroprusside
